# Are the early childhood antecedents of men’s external locus of control similar to those of their female partners?

**DOI:** 10.12688/wellcomeopenres.14098.2

**Published:** 2021-02-02

**Authors:** Jean Golding, Yasmin Iles-Caven, Genette Ellis, Steven Gregory, Stephen Nowicki

**Affiliations:** 1 Centre for Child and Adolescent Health, Population Health Sciences, Bristol Medical School, University of Bristol, Bristol, BS8 2BN, UK; 2 Department of Psychology, Emory University, Atlanta, GA, GA30322, USA

**Keywords:** ALSPAC, Locus of Control, men, childhood, prenatal smoking, parental influences, breast feeding, maternal warmth

## Abstract

**Background:** The concept of locus of control of reinforcement was introduced by Julian Rotter and has been the focus of intense research for nearly half a century.  Surprisingly little research has been directed at clarifying antecedents of locus of control (LOC) orientations in adult men apart from a few small studies. We previously identified a number of independent antecedents associated with women’s LOC, including features of their parents and early childhood. This raised the question as to whether these factors were also associated with the development of LOC in men.

**Methods:** To identify antecedents of LOC orientations in a representative population of women we previously analysed information concerning characteristics of their parents and their own childhood experiences using pregnant women taking part in the Avon Longitudinal Study of Parents and Children (ALSPAC).  Here we use the same design to determine whether their male partners have similar antecedents of LOC orientation. As previously, we use a hypothesis-free exposome technique using all available information on the parents and childhood of the individuals.

**Results:** We show that men had many of the same antecedent characteristics as the women – in particular, their mother’s year of birth and father’s social group, being exposed to cigarette smoke prenatally, starting to smoke regularly before the age of 11, and having a friend die were all associated with being external. Associations of internality common to both were warm maternal care, being breast fed, being born in an area other than that where they currently live, attending boarding school and having a parent admitted to hospital.

**Conclusions:** In general, the antecedents of male external and internal personalities have many similarities to those of women, thus providing some features to inform the possible theoretical background as to how LOC might develop over time.

## Introduction

Locus of control (LOC) refers to individuals’ generalized expectancy regarding the connection between their behavior and its consequences in a problem solving context. Those who fail to see a connection between what they do and what happens to them and instead view what happens to them as the result of luck, fate, chance, or powerful others are seen as externally controlled (ELOC). Conversely those who tend to perceive a connection between their efforts and what happens are called internally controlled (ILOC).

Because of the hundred plus definitions of “locus of control” sprinkled throughout the research literature it is important that each study clearly state and define which LOC concept and measure are being used ( Infurna & Reich, 2016; Skinner, 1996). Peterson & Stunkard (1992) noted the possible problems that could result from using cognates, like efficacy and perceived control ( Bandura, 1986; Infurna & Mayer, 2015; Lachman & Weaver, 1998) or attribution ( Peterson & Seligman, 1984; Seligman, 1975) interchangeably with locus of control of reinforcement as described by Rotter ( Rotter, 1954; Rotter, 1966). He defined locus of control of reinforcement as being a generalized expectancy within his social learning theory ( Rotter, 1954; Rotter, 1966) noting, among other things, that LOC was not a trait. In contrast to traits that characterize people’s personality and operate similarly across situations, Rotter pointed out that a generalized expectancy like LOC should have its greatest impact in situations that are novel, ambiguous or transitory.

Peterson & Stunkard (1992) described how LOC differed from self-efficacy and attribution as follows: “Locus of control refers to one’s generalized expectancies about the origin of rewards and punishment in the world; self-efficacy refers to one’s belief about whether a given behaviour can be enacted, and explanatory style refers to one’s habitual way of explaining the causes of events”.

Although each cognate has generated its own significant and extensive set of findings, it is also true that the findings often overlap one another. That could suggest that they simply are different ways of referring to the same cognate and blurs the reality that the cognates actually can trace their origins to different theoretical perspectives and are measuring something different from one another. In the present study the cognate we are using is the one called locus of control of reinforcement (LOC) that was introduced by Rotter (1966).

Over the past 50 years LOC, as defined by Rotter, has proven to be one of the most frequently studied variables in personality psychology and has been found to be significantly related to an increasing number of important and significant aspects of human life including personality characteristics, social adjustment, academic achievement, health, and business success ( Lefcourt, 1982; Lefcourt, 1983; Nowicki, 2016a; Nowicki & Duke, 2016; Rotter, 1966; Rotter, 1975; Rotter, 1990).

Rotter offered clear theoretical assumptions for the development of LOC expectancies. For him basic LOC orientations are initially learned through children’s experiences with their parents. To facilitate the learning of internal LOC expectancies Rotter suggested that parents: (1) consistently reinforce children’s behavior contingently; (2) allow children more autonomy and independence; and (3) create a nurturing safe environment within which children can discover the connections between how they behave and the consequences.

Carton & Nowicki (1994) reviewed the extant literature to evaluate whether these theorized antecedents of LOC were supported. They concluded that there was empirical support for four parental factors in the development of children’s LOC: (a) The degree of control parents exhibited over their children: more control, higher externality, less control more internality. (b) Externality was associated with a greater degree of life stress produced by father absence due to divorce or death and/or by intense marital discord. (c) Children’s internality was associated with parents who were perceived by children or by themselves as warm, emotionally supportive and nurturing. (d) Internality was associated with parents who rewarded and punished consistently and contingently. However, Carton & Nowicki (1994) also noted that these conclusions were based on data gathered from research studies that used relatively few participants from homogeneous populations of participants, most often children.

Not surprisingly, most studies that have examined “antecedents” of LOC have been focused on children, and the factors associated with their being more internally or externally controlled. Fewer have been interested in identifying child or adolescent precursors of adult LOC, and fewer still have focused on adult males.

Data concerning possible antecedents of LOC in adults would be helpful in obtaining a better understanding of the developmental processes that are associated with internality versus externality throughout the lifespan. With such information, more effective intervention programs could be developed to change or maintain LOC in children and adults.

Data previously analyzed ( Golding *et al.*, 2017a; Golding *et al.*, 2017b) were obtained from pregnant women who were taking part in the Avon Longitudinal Study of Parents and Children (ALSPAC) to determine features of their own parents and their childhood associated with their LOC orientations. Externality was defined as an LOC score that was greater and internality defined as equal to or less than the median score on the Anglicized Nowicki-Strickland Internal-External control scale (ANSIE) ( Nowicki, 2016b; Nowicki & Duke, 1974). This measure was designed to be consistent with Rotter’s (1966) definition of LOC and the test he developed to measure it, but with an easier reading level. Detailed analysis showed that the likelihood of women being external increased with certain details of their parents’ demographic background, features of early and mid-childhood, traumatic events and their social environment ( Golding *et al.*, 2017a; Golding *et al.*, 2017b).

While helpful, these studies are only the first steps in describing the possible events and experiences associated with becoming internal or external in a representative population of adults. In the present study, we took the next step by examining antecedents of LOC in a representative population of male participants to see how similar they were to those identified among their female counterparts. The empirical literature does not offer us much aid in making predictions about gender differences in LOC antecedents so no such predictions were made. Generally then, Rotter suggests that the degree of parental consistency and nurturing plays an important role in determining LOC for both men and women. Schneewind (1997) offers a concise summary of previous parental antecedents that guides our expectation for what we find in the present study.

“Parents providing a stimulating family environment, being consistently and contingently responsive to their children’s behaviour, emphasizing early independence training, engaging autonomy granting, and less intrusive interactions, using less hostile and more inductive disciplinary techniques and relating to the child in a warm and emotionally supportive way, tend to have children with a more internal control orientation. Conversely, parents who provide less stimulation, who are less responsive and more authoritarian, intrusive, overprotective, rejecting, or neglectful are more likely to have children with an external control orientation.”

## Material and methods

### The ALSPAC study

This pre-birth cohort was designed to determine the environmental and genetic factors that are associated with health and development of the study offspring and their parents ( Boyd *et al.*, 2013; Golding *et al.*, 2001). Enrolment in the study was voluntary, but a number of tactics were employed to invite pregnant women with an expected date of delivery between April 1991 and December 1992 to take part. Strategies included encouragement through the local media, general practitioners, midwives, health services and obstetric hospitals; women then contacted the study center for further information, and they were then sent a series of questionnaires to be completed in their own homes. Uniquely among the major UK cohort studies at that time, it was decided to include the fathers of the children. This produced some discussion among the ALSPAC advisory committees, especially as a large proportion of the pregnant women were not married. It was therefore decided to invite the study women to involve their partners if they so wished. To this end questionnaires were sent to her to pass to her partner if she was happy for him to take part. This strategy was approved by the ALSPAC Ethics and Law Committee (ALEC) ( Birmingham, 2018). Consequently there was no immediate way in which the study administrators knew the identity of the study fathers. Given the uncertainty of this approach, it is striking how many of their partners took part during the pregnancy (10,000 compared with 13,867 pregnant women ( Fraser *et al.*, 2013). These are the men studied in this paper. This method by which the men were involved in the study was approved by a number of ethics committees, and the return of self-completion questionnaires continues to be considered acceptable as ‘implied consent’ ( Birmingham, 2018).

Because it was thought that features of the birth of the baby, and any difficulties involved, might alter the parents’ responses in regard to their attitudes and behaviors, there was a concerted effort before the end of the pregnancy to obtain details of their personalities, moods and attitudes, including a measure of their LOC. For full details of all the data collected see the study website. Ethical approval for the study was obtained from the ALSPAC Ethics and Law Committee and the Local Research Ethics Committees ( Birmingham, 2018).

For this project we concentrate on the data collected from questionnaires completed before the birth of the study child. The pregnant women were sent four questionnaires for themselves, and two for their partners during the pregnancy. The LOC scale was included in each parents’ set of questionnaires.

### The outcome measure

The LOC measure used in the present study is a shortened version of the adult version of the Nowicki-Strickland Internal-External locus of control scale (ANSIE) which comprises 40 items in a yes/no format to assess perceived control ( Nowicki & Duke, 1974). This was chosen over other scales more specifically related to perceived control over health, as it was considered that this more generalized scale would relate to other factors in addition to health outcomes. Construct validity for the scale has been found in the results of over a thousand studies ( Nowicki, 2016a). The version used in the present study comprises 12 of the original 40 items which were chosen after factor analysis of the ANSIE administered as a pilot to 135 mothers in the USA. From the responses LOC scores were derived for the men as well as for the women, the higher the score the more external the LOC. The scores ranged from 0 to 12.

The frequency for the women was roughly normally distributed with a median of 4, whereas for the men the distribution was less obviously Gaussian, with a median of 3. For this study external locus of control (ELOC) was defined as having a score greater than the median. This cut-off identified 46.6% of the men as externally controlled.

### The possible antecedent variables considered

In this paper we consider five different groups of variables pertaining to: (a) the demographic background of the parents of the men; (b) their birth and early childhood (< 6 years); (c) features of their mid-childhood (6–11 years) and adolescence (12–15 years); (d) traumatic events occurring during childhood; and (e) their social environment during childhood. The details of the variables are described below. The research questions concern the extent to which different aspects of the backgrounds of their parents and childhoods are associated with the ELOC of the men, and whether these factors are similar to those associated with the ELOC of the women as described elsewhere ( Golding *et al.*, 2017a; Golding *et al.*, 2017b).

***a. Parental education level achieved***

Information was obtained on all the qualifications of the man’s mother and father. From the information obtained a 5-point education scale has been obtained for each, with the following categories: No qualifications; Not higher than CSE or GCSE (D, E, F or G); O-Level or equivalent; A-level or equivalent, such as Teaching or Nursing qualification; University degree. This scale was similar to that derived for the Child Health & Education Study ( Osborn *et al.*, 1984). For the present study, these qualifications have been categorized into two groups: O-level and above; lower than O-level.

***b. Occupations of parents***

Data were obtained concerning the employment situation of each of the parents of the men with details of their normal job, occupation, trade or profession with the type of industry or service given. These occupations were classified using the Standard Occupational Classification (SOC) codes published by the Employment Department Group Office of Population Censuses and Surveys of Great Britain ( Office of Population Censuses & Surveys, 1990). The SOC divides occupations into groups based upon the qualifications and skills necessary to perform each job optimally.

***c. Ethnic origins***

The ethnic origins of the man and each of his parents were obtained using the format asked in the 1991 United Kingdom Census. This categorizes individuals as White, Black/Caribbean, Black/African, Black/Other, Indian, Pakistani, Bangladeshi, Chinese, Other Specified. In the Avon area at this time, only about 6% of the population comprised ethnic minorities.

***d. Home stability***

The man was asked to rate the stability and predictability of each of his parent’s behavior during his childhood. The question was: ‘was your parent’s behavior stable and predictable to you as a child?’ with possible answers: *always, mostly, rarely, never*. The data were obtained for his mother, father, mother figure, father figure, and a ‘home stability’ variable derived from the answers. These questions were created by Karen Thorpe specifically for ALSPAC.

***e. Childhood happiness***

Also developed by Karen Thorpe, the questions were worded: ‘Looking back would you call your childhood happy?’ For each of the ages 0–5, 6–11, 12–15 the man was given options *‘yes very happy’, ‘yes moderately happy’, ‘not really happy’, ‘no, quite unhappy’, ‘no, very unhappy’*. After these questions there was space for any comments he might like to add. From the answers to the three questions, a variable concerning childhood happiness was derived to distinguish those who were very happy throughout from those who were not.

***f. Other data relevant to the three age periods - his early childhood, mid-childhood and adolescence***

Information was obtained on specific persons resident in the household during the three time periods *viz.* mother, father, step-father, step-mother, step-brother, step-sister, mother’s partner.

***g. Childhood life events***

This comprised a set of 30 specific questions administered in mid pregnancy. Childhood was specified as being <17 years. The items were devised by the ALSPAC Study Team based on the earlier work of Coddington (1972). The items included four on deaths to parent(s), relative(s), sibling(s) and friend(s); three on serious illness to the participant as well as to a parent and sibling; three on experiencing a serious accident (to parent, participant, sibling); three on hospitalization (to parent, participant and sibling); three concerning abuse to the participant (physical, sexual and emotional); seven relating to parents (separated, divorced, had serious arguments, remarried, imprisoned, mentally ill, family became poorer); and seven to the participant themselves (discovered that she/he was adopted, failed an important exam, moved to a new district, in trouble with the police, expelled or suspended from school, became physically deformed, girlfriend became pregnant. The study used the responses as to whether each of the different life events had occurred or not.

***h. Other information relating to childhood***

Data collected included the number of schools attended before the age of 16, the number of younger and older siblings, and whether he was a twin or not, as well as whether he had been adopted, taken into care, or had experienced a number of other events during his childhood. Respondents reported whether their parents had divorced before their 16th birthday and the age at which the divorce occurred. Respondents also reported whether their biological mother or father died before their 16th birthday and their (i.e. the respondent's) age when the death occurred.

***i. Relationship with his mother.***

His relationship with his own mother (or mother figure) was elicited using a 22-item set of questions modified from the original Parent-Bonding Instrument (PBI). Respondents reported the quality of their relationship with their mother in childhood on two scales: the care and over protectiveness subscales ( Parker *et al.*, 1979). Previous research supports the validity and reliability of these scales, particularly their association with depression and the validity of retrospective reports ( Parker, 1981).

The original Parent Bonding Instrument ( Parker *et al.*, 1979) had been adapted by Gamsa (1987) to reword the statements that had produced double negatives in the original. During the course of piloting it became obvious that our parents were unhappy with the original options for responses (very like, moderately like, moderately unlike, very unlike) and they have been changed to: ‘never’, ‘sometimes’, ‘usually’. In addition, three questions were omitted since they were almost identical to other questions in the scale and caused considerable annoyance to participants. The introduction to the 22 statements read as follows: ‘we would like to know how you and your mother got on when you were a child. This will probably have varied over your childhood and in different situations but we would like a general impression. Please tick the box to indicate how you mostly remember your mother in the first 16 years’.

Two scores were derived from these 22 questions: a ‘maternal care’ score, and an ‘overprotective’ score. Internal consistencies in this sample were 0.73 and 0.70 for care and over-protectiveness, respectively ( O’Connor *et al.*, 1999). This instrument has been used with the ALSPAC data to show that the link between divorce of one’s parents during childhood and adult depression and / or divorce was partly mediated through the quality of parent-child relations ( O’Connor *et al.*, 1999).

### Statistical analyses

The following exposome analyses were undertaken using STATA version 14 for the ELOC of the study men in the same way as for the study women – i.e. (i) the unadjusted associations with ELOC were calculated for each of the groups of variables; (ii) the variables with unadjusted P value < 0.05 were selected and offered to a backward logistic regression analysis for each group; (iii) the results for each group were considered in regard to the numbers of individuals left in each regression and variables were either dropped or recoded to increase the numbers available in the regression where feasible; (iv) once these intra-domain regressions were finalized, the groups were combined for inter-group analyses in a similar way to our earlier publications ( Golding *et al.*, 2017a; Golding *et al.*, 2017b). Comparison of goodness-of-fit (GOF) between the analyses used 100 times the pseudo-R ^2^ statistic, the higher the value the better the fit.

It should be noted that this is a hypothesis generating study. Consequently to avoid type 2 error, no account is taken of the number of tests undertaken.

## Results

The demographic backgrounds of the individuals in this study are shown in Table S1 ( Supplementary File 1). The LOC score was available for 8645 men, and had a mode and median of 3.

### Relationships with characteristics of his parents

Similar to the study of LOC among the women ( Golding *et al.*, 2017a; Golding *et al.*, 2017b), we considered several different variables describing demographic features of each of the men’s parents; the percentage of men who had an ELOC are depicted in Table 1. This shows that the proportion of the men with ELOC varied with their mother’s year of birth, such that the more recent the mother’s birth the higher the proportion (p<0.0001). The mothers with higher educational qualifications had a substantially lower proportion of sons with ELOC (P<0.0001), but the men were at greater risk of externality if their mothers were aged less than 25 when they were born, if their mothers were smokers, particularly if they had smoked when pregnant with them, or if their mothers had a routine type of occupation (all P<0.0001). There was only a marginal association with the mother’s ethnic background, the 4% of those men with a non-white mother having a lower rate of ELOC (P=0.042).

**Table 1.** Proportion of men with external locus of control (ELOC) and demographic features of their mothers and fathers.

Features of parents		MOTHERS			FATHERS		
		% (n) ELOC	OR [95% CI]	P	% (n) ELOC	OR [95% CI]	P
Year of Birth		N=5341		<0.0001	N= 5127		<0.0001
Pre–1920	}	35.0% (307)	0.85 [0.71, 1.02]		37.0% (241)	0.96 [0.79, 1.18]	
1920–1924					37.9% (266)	1.00 [0.82, 1.21]	
1925–1929		37.3% (335)	0.94 [0.78, 1.12]		37.1% (375)	0.96 [0.81, 1.15]	
1930–1934		38.8% (454)	1.00 Ref		37.9% (412)	1.00 Ref	
1935–1939		42.6% (455)	1.17 [0.99, 1.38]		42.6% (428)	1.47 [1.23, 1.76]	
1940–1944		51.6% (444)	1.68 [1.41, 2.01]		52.5% (287)	1.81 [1.47, 2.22]	
Post 1944		62.7% (294)	2.65 [2.12, 3.30]		65.3% (147)	3.08 [2.28, 4.16]	
Ethnic Group		N=7865		0.042	N=7844		0.273
White		46.4% (3576)	1.00 Ref		46.3% (3544)	1.00 Ref	
Non-white		38.4% (63)	0.72 [0.52, 0.99]		42.3% (83)	0.85 [0.64, 1.13]	
Education Level		N=6022		<0.0001	N=6045		<0.0001
≥O-Level		32.6% (658)	0.51 [0.46, 0.58]		33.2% (765)	0.52 [0.47, 0.58]	
<O-Level		48.5% (1943)	1.00 Ref		48.7% (1824)	1.00 Ref	
Age at Birth of Subject		N=6670		<0.0001	N=6366		<0.0001
<25		49.5% (1152)	1.43 [1.29, 1.59]		49.6% (596)	1.37 [1.20, 1.57]	
25–34		40.7% (1405)	1.00 Ref		41.8% (1487)	1.00 Ref	
35–39	}	40.9% (364)	1.01 [0.87, 1.18]		39.7% (369)	0.92 [0.79, 1.06]	
40+					41.1% (278)	0.97 [0.82,1.15]	
Ever Smoked		N=7829		<0.0001	N=7274		<0.0001
Yes		49.8% (2206)	1.41 [1.29, 1.55]		47.5% (2692)	1.34 [1.20, 1.50]	
No		41.3% (1404)	1.00 Ref		40.3% (648)	1.00 Ref	
Smoked Prenatally		N=7797		<0.0001	-	-	
Yes		52.6% (1694)	1.56 [1.42, 1.71]				
No		41.6% (1904)	1.00 Ref				
Social Group		N=4155		<0.0001	N=7173		<0.0001
Higher managerial		31.0% (35)	0.86 [0.56, 1.31]		28.8% (290)	0.76 [0.64, 0.89]	
Lower managerial		34.4% (277)	1.00 Ref		34.8% (630)	1.00 Ref	
Intermediate		38.4% (385)	1.19 [0.98, 1.44]		43.1% (143)	1.41 [1.12, 1.99]	
Small employers		36.1% (79)	1.08 [0.79, 1.47]		51.6% (393)	1.99 [1.68, 2.36]	
Lower supervisory		51.2% (43)	2.00 [1.27, 3.14]		49.8% (1015)	1.85 [1.63, 2.11]	
Semi-routine		49.3% (544)	1.85 [1.54, 2.23]		53.4% (248)	2.15 [1.75, 2.64]	
Routine		58.9% (487)	2.73 [2.23, 3.34]		61.8% (470)	3.02 [2.53, 3.60]	

OR indicates the unadjusted odds of their son having an ELOC orientation; in brackets are the numbers of men with ELOC.

Logistic regression showed that the major features of his mother that were independently associated with her son’s increased risk of ELOC were poor maternal education, her year of birth (the more recently she had been born the higher the risk), her social group (the less professional the higher the risk) and whether she smoked when pregnant ( Table S2; Supplementary File 1). In the presence of these factors, the age of the mother at the birth of her son, and her overall smoking history failed to enter. However, due to the relatively small number of mothers for whom the social group had been recorded, the total numbers in this regression (Model A) were only 2258 with a GOF of 4.2. Omitting the social group variable resulted in an increase in numbers to 4067 but a slight reduction in GOF to 3.8. Because of the increase in numbers, and relatively small reduction in the GOF, we have retained this version in the rest of this paper ( Table S2; Supplementary File 1). Thus model (B) comprised his mother’s education level, her year of birth and whether she smoked when pregnant with him (all P<0.0001).

For his father, unadjusted relationships showed significant associations with his father’s year of birth, his father’s education level, whether he was aged <25 at the birth of his son, whether he had a history of smoking as well as his social group (all P<0.0001). Intra-domain logistic regression indicated that the major independent factors predictive of ELOC comprised his father’s year of birth, level of education, history of smoking and his social group, the more routine the occupation the more likely the son to have an ELOC. The father’s age was attenuated by other factors in this model, which included 3578 observations and had a GOF of 4.4 ( Table S3; Supplementary File 1).

Combining the information from both parents resulted in a model with just five variables: the year of birth of each parent, his mother’s education, his father’s social group and whether his mother had smoked when pregnant with her son. In the presence of these variables the father’s history of smoking and his education level did not enter ( Table 2).

**Table 2.** Backwards step-wise logistic regression of the man’s locus of control score (≥4 versus <4): his parents).

	Intra domain		
Variable	N	P	OR [95% CI]
Mother’s education ≥ O-Level	3652	<0.001	0.75 [0.64, 0.87]
Mother’s year of birth ^a^	3652	<0.001	1.27 [1.10, 1.47]
Mother smoked when pregnant	3652	<0.0001	1.41 [1.22, 1.62]
Father’s year of birth ^b^	3652	0.032	1.15 [1.01, 1.31]
Father’s social group ^c^	3652	<0.0001	1.17 [1.12, 1.21]

Total N=3652, Goodness of fit measure =4.73 ^a^pre 1925; 1925–1939; 1940–1944; post 19; ^b^pre 1935; 1935–1939; 1940–1944; post 1944. ^c^the seven categories

### Relationships with facets of his early childhood

Of the 16 variables recorded relating to his early childhood (<6 years), eight were statistically significantly related to ELOC before adjustment ( Table 3): they comprised an increase in risk if step-father, father’s partner or step-sibling was present in the home, whether he had two or more older siblings, whether his parents had divorced or separated during this period and whether he described this period of his life as unhappy. Conversely, if he had been breast fed, or was born to parents residing outside the Avon area, he was more likely to have an internal LOC.

**Table 3.** Associations between features of early childhood (≤5yrs) and external locus of control (ELOC).

Features of his childhood			
	%(n) ELOC	OR [95% CI]	P
***In First Year***			
Ethnic Background	N=8292		0.239
White	46.1% (3731)	1.00 Ref	
Non-white	41.8% (82)	0.84 [0.63, 1.12]	
Place of Birth	N =7856		<0.0001
Avon	56.3% (2616)	1.00 Ref	
Rest of England	31.8% (776)	0.36 [0.33, 0.40]	
Rest of World	36.7% (282)	0.38 [0.35, 0.42]	
Was Adopted	N=8768		0.705
Yes	48.5% (49)	1.08 [0.73, 1.60]	
No	46.6% (4041)	1.00 Ref	
Was Breastfed	N=5027		<0.0001
Yes	40.2% (1351)	0.62 [0.55, 0.70]	
No	52.0% (867)	1.00 Ref	
No. of Older Siblings	N=4850		<0.0001
0	36.6% (704)	1.00 Ref	
1	39.7% (619)	1.14 [1.00, 1.31]	
2	44.5% (354)	1.39 [1.17, 1.64]	
3+	50.6% (289)	1,78 [1.47, 2.15]	
***In First 5 Years***			
Mother Present in Home	N=8768		0.905
Yes	46.6% (3819)	0.99 [0.84, 1.17]	
No	46.9% (271)	1.00 Ref	
Father Present in Home	N=8768		0.097
Yes	46.4% (3706)	0.88 [0.76, 1.02]	
No	49.5% (384)	1.00 Ref	
Step-father Present in Home	N=8768		<0.001
Yes	68.8% (53)	2.55 [1.57, 4.13]	
No	46.5% (4037)	1.00 Ref	
Step-brother Present in Home	N=8768		0.098
Yes	56.9% (37)	1.52 [0.93, 2.48]	
No	46.6% (4053)	1.00 Ref	
Step-sister Present in Home	N=8768		0.008
Yes	63.9% (39)	2.04 [1.21, 3.44]	
No	46.5% (4051)	1.00 Ref	
Mother’s Partner Present	N=8768		0.282
Yes	55.9% (19)	1.45 [0.74, 2.86]	
No	46.6% (4071)	1.00 Ref	
Father’s Partner Present	N=8768		0.029
Yes	71.4% (15)	2.87 [1.11, 7.39]	
No	46.6% (4075)	1.00 Ref	
Parents Divorced/ Separated	N=8768		<0.0001
Yes	59.6% (167)	1.72 [1.35, 2.19]	
No	46.2% (3923)	1.00 Ref	
Mother Died	N=8768		0.783
Yes	48.4% (30)	1.07 [0.65, 1.77]	
No	46.6% (4060)	1.00 Ref	
Father Died	N=8768		0.268
Yes	51.7% (62)	1.23 [0.85, 1.76]	
No	46.6% (4028)	1.00 Ref	
Recollection of Age ≤5 Years	N=7306		<0.0001
Very happy	45.4% (2598)	1.00 Ref	
Moderately happy	52.2% (737)	1.31 [1.17, 1.47]	
Not really happy	60.7% (68)	1.86 [1.27, 2.73]	
Quite unhappy	75.0% (15)	3.61 [1.31, 9.95]	
Very unhappy	73.0% (27)	3.25 [1.57, 6.72]	

(In brackets are the numbers of men with an external orientation)

For the logistic regression we omitted the variable concerning presence of father’s partner as there were only small numbers involved. Backwards stepwise logistic regression using the remaining seven factors showed that only three remained (being born outside Avon, being breast fed and the number of older siblings, the latter being of borderline significance (P = 0.034; Table S4, Model A; Supplementary File 1). However the numbers left in this model were small (2767), so in a further exercise we dropped the older siblings variable (which was responsible for the decrease in numbers) and reanalyzed. This resulted in larger numbers in the model (4056) and an increase in the GOF (from 3.83 to 4.72). This analysis also indicated that the step-father and step-sibling effects were attenuated by other features (parents had separated or divorced and the man’s recollection of unhappiness in this part of childhood); thus four variables remained: born to parents who resided outside Avon [OR 0.40, 95% CI 0.35, 0.45, P<0.0001]; having been breast fed [OR 0.68, 95% CI 0.59, 0.78, P< 0.001]; parents divorced or separated [OR 1.93; 95% CI 1.27, 2.92; P=0.002]; and the degree of unhappiness in early childhood recalled by the man [OR 1.21, 95% CI 1.05, 1.38, P = 0.006] ( Table S4, Model B; Supplementary File 1).

### Mid-childhood and adolescence

The ways in which the odds of a man having an ELOC varied for features of mid-childhood (ages 6–11) and adolescence (age 12–16) are shown in Table 4. The strongest positive associations concerned parents having divorced or separated, step-siblings or step-father being present and the degree of unhappiness felt during each time period. In contrast there were protective effects for presence of the biological father in the home. The presence of his mother at these time points appeared to be of marginal impact. Logistic regression involving the group of eight variables with unadjusted P < 0.05 identified for mid-childhood showed that just four remained in the model predicting ELOC – presence of the father (negative) and mother’s partner (positive), degree of unhappiness and having started smoking regularly before age 11 ( Table S5a; Supplementary File 1). For the 10 such variables identified in adolescence five remained in the model: father present in the household (protective), and presence of brother(s) or sister(s), degree of unhappiness and starting to smoke regularly ( Table S5b; Supplementary File 1).

**Table 4.** Associations between features of mid-childhood (6–11 yrs) and adolescence (12–15 yrs) and external locus of control (ELOC).

Features of the father		MID CHILDHOOD			ADOLESCENCE		
*At ages 6 –11 Years*		%(n) ELOC	OR [95% CI]	P	%(n) ELOC	OR [95% CI]	P
Mother Present in Home		N=8768		0.021	N=8768		0.160
Yes		46.3% (3708)	0.84 [0.72, 0.97]		46.4% (3728)	0.90 [0.77, 1.04]	
No		50.7% (382)	1.00 Ref		49.1% (362)	1.00 Ref	
Father Present in Home		N=8768		<0.0001	N=8768		<0.0001
Yes		45.3% (3416)	0.68 [0.61, 0.77]		45.1% (3282)	0.69 [0.62, 0.77]	
No		54.8% (674)	1.00 Ref		54.4% (808)	1.00 Ref	
Step-father Present in Home		N=8768		<0.0001	N=8768		<0.0001
Yes		60.0% (144)	1.74 [1.34, 2.26]		57.2% (210)	1.56 [1.26, 1.93]	
No		46.3% (3946)	1.00 Ref		46.2% (3880)	1.00 Ref	
Step-brother Present in Home		N=8768		<0.001	N=8768		0.026
Yes		64.0% (64)	2.05 [1.36, 3.09]		55.7% (83)	1.45 [1.04, 2.01]	
No		46.4% (4026)	1.00 Ref		46.5% (4007)	1.00 Ref	
Step-sister Present in Home		N=8768		<0.0001	N=8768		0.009
Yes		69.1% (67)	2.58 [1.67, 3.98]		58.3% (74)	1.61 [1.13, 2.29]	
No		46.4% (4023)	1.00 Ref		46.5% (4016)	1.00 Ref	
Mother’s Partner Present		N=8768		<0.0001	N=8768		0.002
Yes		71.2% (52)	2.86 [1.72, 4.75]		61.0% (75)	1.80 [1.25, 2.59]	
No		46.4% (4038)	1.00 Ref		46.4% (4038)	1.00 Ref	
Parents Divorced/Separated		N=8768		<0.0001	N=8768		<0.0001
Yes		62.0% (259)	1.92 [1.57, 2.35]		58.5% (176)	1.64 [1.30,2.07]	
No		45.9% (3831)	1.00 Ref		46.2% (3914)	1.00 Ref	
Started Smoking Regularly		N = 8525		<0.0001	N = 8525		<0.0001
Yes		74.2% (95)	3.34 [2.24, 4.97]		64.9% (524)	2.27 [1.95,2.64]	
No		46.3% (3887)	1.00 Ref		44.8% (3458)	1.00 Ref	
Recollection of Happiness		N=8358		<0.0001	N=8453		<0.0001
Very happy		45.0% (2353)	1.00 Ref		45.6% (1880)	1.00 Ref	
Moderately happy		49.6% (1272)	1.20 [1.10, 1.32]		48.0% (1522)	1.10 [1.00, 1.21]	
Not really happy		60.7% (227)	1.89 [1.52, 2.34]		52.3% (386)	1.31 [1.12, 2.53]	
Quite unhappy	}	71.1% (135)	3.00 [2.18, 4.13]	{	57.2% (139)	1.60 [1.23, 2.07]	
Very unhappy					69.9% (121)	2.78 [2.00, 3.87]	

### Combining the three age groups

Since there is much overlap between the factors entering the models for each age group (e.g. unhappiness appears in each, presence of the father in two), the surviving variables from each age group were offered together. The results are shown in Table 5 and Table S6 ( Supplementary File 1). This demonstrated that the degree of unhappiness was particularly relevant if occurring in mid-childhood, but that presence of the father was important both in mid-childhood and adolescence. Starting regular smoking was important at both these time points. These findings did not explain the associations with being breast fed or with being born within the study area (Avon), both of which remained in the model.

**Table 5.** Model combining features of early childhood, mid-childhood and adolescence independently predicting a man has an external LOC.

Childhood	OR [95% CI]	P
*Early Childhood*		
Born in Avon	2.56 [2.22,2.86]	<0.0001
Breast fed	0.69 [0.60,0.79]	<0.0001
*Mid-Childhood*		
Father present	0.68 [0.52,0.90]	0.006
Mother’s partner present	2.47 [1.08,5.66]	0.033
Degree of unhappiness	1.26 [1.15,1.39]	<0.0001
Started smoking regularly	3.76 [1.93,7.30]	<0.0001
*Adolescence*		
Father present	0.76 [0.59,0.98]	0.031
Brother present	1.16 [1.02,1.32]	0.020
Started smoking regularly	2.13 [1.71,2.66]	<0.0001

N = 4498; GOF 7.16

OR indicates the adjusted odds of having an ELOC orientation

### His social environment in childhood

A number of other aspects of his childhood were compared with his subsequent ELOC ( Table 6). These included measures indicating contact with social, health and educational care of various sorts – such as being taken “into care”, living with foster parents, living in a children’s home, being taken into custody, seeing a child psychiatrist, having speech therapy and attending a “special school”, all of which were associated with increased risk of subsequent ELOC, as were other residential arrangements such as living with grandparents, other relatives or friends. The only residential category that indicated a LOC advantage concerned the men who had gone to a boarding school. In addition it can be seen from this table that there is a strong protective association with his rating of his mother’s warmth.

**Table 6.** Associations between features of the social environment in childhood and external locus of control (ELOC) for the men.

Social Environment in childhood	%(n) ELOC	OR [95% CI]	P
Attended a special school	N=8768		<0.0001
Yes	70.1% (176)	2.76 [2.10, 3.63]	
No	46.0% (3914)	1.00 Ref	
Saw a child psychiatrist	N=8768		<0.001
Yes	56.8% (167)	1.53 [1.21, 1.93]	
No	46.3% (3923)	1.00 Ref	
Had speech therapy	N = 8768		0.007
Yes	54.4% (160)	1.38 [1.09, 1.74]	
No	46.4% (3930)	1.00 Ref	
Was “in care”	N = 8299		<0.0001
Yes	61.7% (150)	1.83 [1.40,2.37]	
No	46.9% (3779)	1.00 Ref	
Lived with grandparents	N=8490		<0.0001
Yes	57.8% (284)	1.57 [1.31, 1.89]	
No	46.6% (3727)	1.00 Ref	
Lived with other relatives	N=8490		<0.0001
Yes	59.9% (209)	1.70 [1.37, 2.12]	
No	46.7% (3802)	1.00 Ref	
Lived with friends	N=8490		<0.0001
Yes	59.1% (182)	1.64 [1.30, 2.07]	
No	46.8% (3829)	1.00 Ref	
Lived with foster parents	N=8490		<0.001
Yes	68.1% (49)	2.40 [1.46, 3.94]	
No	47.1% (3962)	1.00 Ref	
Went to boarding school	N=8379		<0.0001
Yes	26.9% (186)	0.57 [0.47, 0.68]	
No	47.4% (3764)	1.00 Ref	
Stayed in children’s home	N = 8352		<0.0001
Yes	66.9% (91)	2.28 [1.59, 3.27]	
No	47.0% (3861)	1.00 Ref	
Stayed in custody	N=8325		<0.0001
Yes	83.3% (169)	5.75 [3.97, 8.33]	
No	46.4% (3767)	1.00 Ref	
Left home before age 18	N = 8443		<0.0001
Yes	53.9% (856)	1.39 [1.25, 1.55]	
No	45.7% (3129)	1.00 Ref	
Stability of mother in household	N=8298		<0.0001
Always	46.4% (2225)	1.00 Ref	
Mostly	48.1% (1520)	1.07 [0.98, 1.17]	
Rarely / never	65.4% (223)	2.18 [1.73, 2.75]	
Stability of father in household	N=7940		<0.0001
Always	44.5% (1938)	1.00 Ref	
Mostly	46.9% (1408)	1.10 [1.01, 1.21]	
Rarely /never	66.0% (382)	2.42 [2.02, 2.91]	
Overall stability of home	N=8438		<0.0001
Very stable	46.3% (1834)	1.00 Ref	
Fairly stable	46.0% (1686)	0.99 [0.90, 1.08]	
Unstable	58.7% (338)	1.65 [1.38, 1.97]	
Very unstable	77.2% (183)	3.93 [2.88, 5.36]	
Maternal care score ^a^	N=8768		<0.0001
<19	57.2% (1094)	2.00 [1.74, 2.30]	
19–21	49.2% (870)	1.46 [1.26,1.68]	
22–23	42.5% (1573)	1.11 [0.98, 1.26]	
24	40.0% (553)	1.00 Ref	

^a^the higher the score the warmer the care.

When all 16 of these variables were offered to a backwards stepwise regression, nine were eliminated including being “in care”, living with grandparents, other relatives, friends or in a children’s home, seeing a child psychiatrist or speech therapist; the instability of each of the parents were eliminated in favor of the variable combining the two into the stability of the home. The strongest predictors from the remaining group were being taken into custody, attending a special school, leaving home in childhood, and having an unstable home. In contrast there were strong protective associations with going to a boarding school and of his rating of his mother’s warmth ( Table S7; Supplementary File 1).

### Traumatic events in childhood

The unadjusted relationships between the so-called life events that had occurred in childhood are shown in regard to the risk of ELOC in adulthood in Table 7. It can be seen that some events are positively associated (e.g. death of parent, sibling or friend; had a serious accident, his partner became pregnant, a parent being imprisoned, being physically or emotionally abused, parents separated, divorced, remarried or had serious arguments, he was in trouble with the police, was suspended from school and his family’s finances deteriorated). However there were other events which appeared to be associated with a more internal orientation in adulthood. These included death of a relative, a parent or a sibling being in hospital, he himself being admitted to hospital, and moving to a new district.

**Table 7.** Unadjusted associations between experience of life events in childhood and external locus of control (ELOC).

Experiences in childhood			
	%(n) ELOC	OR [95% CI]	P
A parent died	N=8062		0.010
Yes	51.7% (243)	1.28 [1.06, 1.54]	
No	45.6% (3459)	1.00 Ref	
A sibling died	N=8062		0.005
Yes	54.3% (145)	1.42 [1.11, 1.81]	
No	45.6% (3557)	1.00 Ref	
A relative died	N = 8062		<0.0001
Yes	43.7% (2213)	0.79 [0.72, 0.86]	
No	49.6% (3557)	1.00 Ref	
A friend died	N = 8062		0.002
Yes	49.2% (850)	1.18 [1.06,1.31]	
No	45.0% (2852)	1.00 Ref	
Parent seriously ill	N=8062		0.212
Yes	44.7% (836)	0.94 [0.84, 1.04]	
No	46.3% (2866)	1.00 Ref	
Parent in hospital	N=8062		<0.0001
Yes	42.6% (1464)	0.79 [0.73, 0.87]	
No	48.4% (2238)	1.00 Ref	
Was seriously ill	N=8062		0.766
Yes	45.4% (394)	0.98 [0.85, 1.13]	
No	46.0% (3308)	1.00 Ref	
Was admitted to hospital	N=8062		<0.0001
Yes	43.1% (1422)	0.83 [0.76, 0.90]	
No	47.8% (2280)	1.00 Ref	
A sibling was seriously ill	N=8062		0.823
Yes	45.6% (406)	0.98 [0.86, 1.13]	
No	46.0% (3296)	1.00 Ref	
A sibling was in hospital	N = 8062		0.005
Yes	43.2% (865)	0.86 [0.78,0.96]	
No	46.8% (2837)	1.00 Ref	
Parent had a serious accident	N=8062		0.539
Yes	47.2% (246)	1.06 [0.89, 1.26]	
No	45.8% (3456)	1.00 Ref	
Had a serious accident	N=8062		<0.001
Yes	52.4% (383)	1.33 [1.14, 1.55]	
No	45.3% (3319)	1.00 Ref	
Partner became pregnant	N=8062		<0.0001
Yes	68.0% (155)	2.57 [1.94, 3.40]	
No	45.3% (3547)	1.00 Ref	
A parent was imprisoned	N = 8062		<0.0001
Yes	76.0% (73)	3.79 [2.37, 6.07]	
No	45.6% (3629)	1.00 Ref	
Was physically abused by parent	N = 8062		<0.0001
Yes	58.9% (224)	1.74 [1.41,2.14]	
No	45.3% (3478)	1.00 Ref	
Parents separated	N=8062		<0.0001
Yes	58.2% (690)	1.79 [1.58, 2.03]	
No	43.8% (3012)	1.00 Ref	
Parents divorced	N=8062		<0.0001
Yes	58.8% (569)	1.81 [1.58, 2.074]	
No	44.2% (3133)	1.00 Ref	
A parent remarried	N=8062		<0.0001
Yes	58.6% (441)	1.75 [1.51, 2.04]	
No	44.6% (3261)	1.00 Ref	
Was emotionally abused by parent	N = 8062		0.019
Yes	50.7% (278)	1.23 [1.03, 1.46]	
No	45.6% (3424)	1.00 Ref	
Parents had serious arguments	N = 8062		<0.001
Yes	48.9% (1216)	1.19 [1.08,1.31]	
No	44.6% (2486)	1.00 Ref	
Was sexually abused	N=8062		0.479
Yes	49.5% (48)	1.16 [0.77, 1.72]	
No	45.9% (3654)	1.00 Ref	
A parent was mentally ill	N = 8062		0.778
Yes	45.1% (134)	0.97 [0.77,1.22]	
No	45.9% (3568)	1.00 Ref	
Discovered was adopted	N=8062		0.009
Yes	55.9% (95)	1.50 [1.11, 2.04]	
No	45.7% (3607)	1.00 Ref	
Moved to a new district	N=8062		<0.0001
Yes	41.5% (1097)	0.77 [0.70, 0.84]	
No	48.1% (2605)	1.00 Ref	
In trouble with police	N=8062		<0.0001
Yes	63.6% (934)	2.42 [2.15, 2.72]	
No	42.0% (2768)	1.00 Ref	
Was suspended from school	N = 8062		<0.0001
Yes	71.1% (523)	3.20 [2.71, 3.78]	
No	43.4% (3179)	1.00 Ref	
Family finances deteriorated	N = 8062		0.001
Yes	50.2% (618)	1.22 [1.08,1.38]	
No	45.1% (3084)	1.00 Ref	

Before offering these variables for further analyses we decided to omit the variable concerning his partner becoming pregnant since it was felt that this was likely to be an outcome of his own external orientation rather than being on a causal pathway. Stepwise adjustment of these variables for one another revealed 12 to be independently associated; those positively related to ELOC were deaths of a parent, sibling or friend, having a serious accident, being physically abused by a parent, imprisonment of a parent, parents separating and discovering he was adopted; factors negatively associated with ELOC were death of a relative, hospitalization of himself and/or a parent, and moving to a new district ( Table S8; Supplementary File 1).

### Combining the traumatic and social environment models

The 12 variables concerning the traumatic events ( Table S9; Supplementary File 1) were offered to stepwise regression to the seven from the social environment model. Only two dropped out – discovering he was adopted and being physically abused by his parents.

### Final models

All variables concerning the man’s childhood which had survived the intra-domain analyses were offered together. Of the 26 variables, 15 were left in the model ( Table S10; Supplementary File 1 and Model A in Table 8). The features remaining in the model included the following with protective associations: being born outside the study area (OR 0.40), his father being present in mid-childhood (0.64), being breast fed (0.72), attending boarding school (0.72), positive view of the warmth of his mother’s care of him (0.78), hospitalization of a parent (0.82) or of himself (0.84); in contrast the following variables were positively associated with ELOC: starting to smoke before the age of 11 (3.77), spending time in custody (3.02), mother’s partner being present in the home in mid-childhood (2.89), starting to smoke regularly in adolescence (2.09), attending a special school (1.83), unstable home (1.60), having a serious accident (1.31), and a friend dying (1.17).

**Table 8.** Final models concerning childhood (Model A), and combined with parental characteristics (Model B) predicting the man’s ELOC.

FEATURE	Model A		Model B	
	OR [95% CI]	P	OR [95% CI]	P
*In Infancy*				
Born outside Avon	0.40 [0.35,0.45]	<0.0001	0.51 [0.43,0.61]	<0.0001
Was breast fed	0.72 [0.62,0.82]	<0.0001	0.77 [0.65, 0.91]	0.003
*In Mid-Childhood*				
Father present	0.64 [0.51,0.79]	<0.0001	DNE	
Mother’s partner present	2.89 [1.19,7.02]	0.019	DNE	
Smoked regularly	3.77 [1.87,7.60]	<0.001	4.24 [1.63,11.0]	0.003
*Adolescence*				
Started smoking regularly	2.09 [1.66,2.62]	<0.0001	1.77 [1.32,2.38]	<0.001
*Traumatic Events*				
Friend died	1.17 [1.00,1.37]	0.046	1.23 [1.02,1.49]	0.032
Parent hospitalized	0.82 [0.72,0.93]	0.003	DNE	
Admitted to hospital	0.84 [0.74,0.96]	0.013	DNE	
Had serious accident	1.31 [1.04,1.65]	0.023	DNE	
*Social Environment*				
Attended special school	1.83 [1.23,2.73]	0.003	1.93 [1.18,3.15]	0.009
Went to boarding school	0.72 [0.54,0.95]	0.022	DNE	
Spent time in custody	3.02 [1.71,5.32]	<0.001	2.53 [1.22,5.23]	0.012
Home described as unstable	1.60 [1.33,1.93]	<0.0001	1.76 [1.38,2.25]	<0.0001
Maternal care score	0.78 [0.72,0.85]	<0.0001	0.78 [0.71,0.87]	<0.0001
*Characteristics of his parents*				
Mother’s year of birth	-		1.32 [1.19,1.47]	<0.0001
Mother smoked prenatally	-		1.20 [1.02,1.42]	0.029
Father’s social group	-		1.16 [1.11,1.21]	<0.0001
**Number in model**	**4369**		**2939**	
**GOF**	**9.22**		**9.87**	

DNE = Did not enter; GOF = Goodness of fit

When the three characteristics of the parents were offered to the factors remaining in Model A, a number of the descriptors of childhood dropped out. These included: presence of the father and of the mother’s partner, whether a parent, or he himself was hospitalized in childhood, or he had a serious accident, or went to boarding school. The final model showed an increase in GOF from 9.22 to 9.87, but the number of participants in the model dropped from 4369 to 2939 ( Table 8 and Table S11; Supplementary File 1).

### Summary of the antecedents for men and women

The ways in which the different results from each of the intra-domain models contributed to the final models is shown in Table 9. In general the GOF results show that amalgamation of each of the domains has increased the GOF. However it is notable that the early childhood variables (EC) appear to be less, and the traumatic events (TE) and adolescence (A) variables more important among the men than was previously shown for the women ( Golding *et al.*, 2017b). Overall there were fewer variables in the Final model for the men (12) than the women (20), but the GOF was greater for the men (9.87 v 8.37).

**Table 9.** Summary of goodness of fit (GOF) in models concerning childhood and parental characteristics, comparing results for men and women.

Model	MAN			WOMAN		
	N	No. variables	GOF	N	No. variables	GOF
EC	4056	4	4.72	8614	6	5.79
MC	8188	4	1.78	12,090	5	1.99
A	8279	5	2.21	12,574	3	1.26
EC+MC+A	4498	9	7.16	8945	10	6.14
TE	8062	12	2.34	11,843	10	1.94
SE	7957	7	3.19	10,851	11	2.84
All childhood	4369	15	9.22	8673	22	7.50
M	4067	3	3.83	10,642	4	4.34
F	3578	4	4.38	8110	5	4.64
M+F	3652	5	4.73	5975	9	5.43
ALL	2939	12	9.87	7285	20	8.37

A= Adolescence; EC = Early Childhood (<6y); F = Father; M = Mother; MC = Mid-childhood (6–11y); SE = Social Environment; TE = Traumatic Events; GOF = Goodness of fit measure

A comparison of the results for each item is shown for the men and women in Table 10 using the two final models involving the childhood circumstances. We do not show the results incorporating the demographic features of the parents as the numbers reduced substantially in this model for the men; however we indicate the variables that drop out of the model when the parental demographic features are introduced. The table highlights the results in common between the men and the women. These include associations between ELOC and starting to smoke regularly before the age of 11, and having a friend die. Associations with increased internality common to both were warm maternal care, being breast fed, being born in an area other than that where they currently live (Avon), attending boarding school and having a parent admitted to hospital.

**Table 10.** Comparison of childhood features in the final model for men and women.

Childhood characteristic	Men (n=4369)	Women (n=8675)
*In Early Childhood*		
Born outside Avon	0.40 [0.35, 0.45]	0.55 [0.50, 0.61]
Was breast fed	0.72 [0.62, 0.82]	0.87 [0.79, 0.96] ^*^
Had a birthmark	-	1.19 [1.07, 1.32] ^*^
No. older siblings	-	1.26 [1.12, 1.41]
Father present	-	0.62 [0.51, 0.76]
Year of birth	-	1.53 [1.43, 1.65]
*In Mid-childhood*		
Father present	0.64 [0.51, 0.79] ^*^	-
Mother’s partner present	2.89 [1.19, 7.02] ^*^	-
Smoked regularly	3.77 [1.87, 7.60]	1.72 [1.06, 2.78]
Degree of happiness	-	0.88 [0.81, 0.95]
*In Adolescence*		
Started smoking regularly	2.09 [1.66, 2.62]	-
Mother present	-	0.79 [0.63, 1.00] ^*^
*Social Environment*		
Maternal care score	0.78 [0.72, 0.85]	0.77 [0.72, 0.84]
Boarding school	0.72 [0.54, 0.95] ^*^	0.46 [0.35, 0.61]
Home was unstable	1.60 [1.33, 1.93]	-
Attended special school	1.83 [1.23, 2.73]	-
Was in custody	3.02 [1.71, 5.32]	-
Attended child psychiatrist	-	1.36 [1.06, 1.76] ^*^
Lived with grandparents	-	1.40 [1.11, 1.76]
Left home before aged 18	-	1.41 [1.24, 1.59]
Stayed elsewhere	-	0.78 [0.64, 0.95] ^*^
*Traumatic Events*		
Friend died	1.17 [1.00, 1.37]	1.21 [1.07, 1.39]
Relative died	-	0.85 [0.77, 0.93]
Parent hospitalized	0.82 [0.72, 0.93] ^*^	0.90 [0.82, 0.99] ^*^
Sibling hospitalized	-	0.84 [0.75, 0.94]
Parent had serious accident	-	1.45 [1.16, 1.82] ^*^
Was hospitalized	0.84 [0.74, 0.96] ^*^	-
Had serious accident	1.31 [1.04, 1.65] ^*^	-
Physically abused by parent	-	0.73 [0.55, 0.98] ^*^
Parent was mentally ill	-	0.65 [0.51, 0.82]

*Did not enter when features of the parents were entered

## Discussion

Men and women appear to have more in common with one another than not when it comes to antecedents of their loci of control; that is suggested by our results. At least in terms of the characteristics included in this study, men and women appear to have a common core of experiences such as maternal warmth (as reflected in the maternal care scores, and being breast fed), consistency (as shown by a stable home), and greater satisfaction with life (as shown by a lower rate of unhappiness) that may underlie a tendency to be more internal than external for both men and women. Antecedents like these describe a home situation that provides what is needed for children to feel comfortable and safe enough to explore their environments and to learn more about the contingencies existing between their behavior and outcomes.

Since maternal warmth was found to be important for internality and lack of it for externality in both men and women, it is apparent that efforts to strengthen the mother-son relationship might pay off in increased likelihood of internality in men as well as in women. Several authors ( Carton & Nowicki, 1994; Rotter, 1966; Rotter, 1975; Rotter, 1990; Schneewind, 1997) have indicated that the tone of the mother-son relationship sets the stage for initially learning how to interact with the environment such that if the relationship is positive and supportive, it provides the child with a comfortable vantage point to become aware of his behavioral impact.

However, while mothers’ warmth was significant for men, it also may be true that an additional protective factor for them was the presence of the biological father in the home, especially in mid-childhood. The presence of the father may strengthen the stability of the family and, in some cases, present the male child with an accessible same sex model of internality if that is the father’s orientation. Further study is needed to see if fathers who stay with the family are more likely to be internal; conversely if they are external, how might that affect the development of LOC in their sons. In any case, this finding suggests that gaining additional insight into the father-son relationship could be helpful in identifying significant factors associated with the development of internal or external control expectancies in the sons.

One additional difference deserves some comment and that is the fact that all participants born outside of Avon were likely to be more internal than their peers. How does that fit in with the maternal warmth, home stability, and the presence of the father? One possible explanation may be that if sons who have a warm and stable household move to live in a different location, the change would give them a greater number of environmental opportunities to learn contingencies necessary for the development of internality. The same reasoning is possible for the apparent benefits of attending boarding school. If true, it suggests that a strong, stable, family situation may provide an important foundation for reacting positively when experiencing change in living situations. It is also possible that the opposite is true. That is, if the family situation lacks warmth and is unstable then moving to a new environment might be overwhelming and facilitate the development of externality.

### Smoking exposures

Previous studies ( Golding *et al.*, 2017a; Golding *et al.*, 2017b) had shown that women who were externally oriented had an increased risk of (a) having been exposed *in utero* to their mother’s smoking, and (b) being a regular smoker themselves by the age of 11. Similarly, the same smoking factors were demonstrated for the men ( Table 8) with adjusted odds ratios of 1.20 [95%CI 1.02, 1.42] and 3.77 [1.87, 7.60] respectively. These factors were independently associated and were not explained by social conditions. This raises the question as to whether mothers who smoke are themselves more externally oriented, and hence more likely to permit their offspring to smoke in mid-childhood or whether childhood exposure to cigarette smoke has a biological effect on the developing brain resulting in susceptibility to ELOC. While it is most likely that there are psychological and social reasons for maternal prenatal smoking to be associated with ELOC, a biological effect is plausible: animal experiments find that fetal exposure to a smoking mother results in an increased risk of poor neurocognitive functioning, results that have been mirrored by observational studies in humans ( Bublitz & Stroud, 2012; Cornelius *et al.*, 2011; Wickström, 2007). Brain imaging techniques have demonstrated reductions in grey matter volume and density among smokers ( Dome *et al.*, 2010), suggesting a biological effect since the grey matter includes regions of the brain involved in memory, decision making, and self-control and two imaging studies have demonstrated an association between hippocampal volume and LOC in young adults ( Miller & Alston, 2008; Pruessner *et al.*, 2005).

It is feasible that some of the features that we have shown to be associated with LOC are a consequence of the externality of the individuals in childhood. This could explain the strong association with starting to smoke by age 11, but cannot explain the associations with prenatal exposure – although this may be the result of having an externally oriented mother. However, the correlation between the LOC orientation of mothers and their children is generally low so may not be a plausible interpretation ( Schneewind, 1997).

### Strengths of the study

The major strengths of the study are the large numbers of individuals involved, the fact that they were selected from a geographical population, and that the questions asked of both the men and women considering their backgrounds were identical. Both the men and women were asked at the same point in their life cycle – i.e. when about to become a parent – although the men were, on average, 3 years older than the women.

### Limitations of the study

There are a number of limitations to this study, the most important of which is likely to be the amount of missing data concerning the childhood of the men compared with the women. This is a general observation – that men are far less interested in their backgrounds and family history than are women. This is particularly true of men who had little contact with their fathers, and who are particularly likely to drop out of any model. The non-responders were also likely to be weighted with ELOC individuals, and were likely to fall within the category ‘missing not at random’. We therefore decided not to impute the missing data ( Sterne *et al.*, 2009).

A further limitation of both the study of the women and the men concerns the Parent Bonding Index ( Parker *et al.*, 1979), one trait within which was shown to be strongly protective for both the men and women (maternal care). For reasons of space on the questionnaires, the individuals were only asked to complete the relevant questions in regard to their relationship with their mothers, not their fathers. In retrospect this was unfortunate since presence of the father was in the final model of both men and women, albeit with the focus at different time points (women in early childhood; men in mid-childhood).

Finally it must be admitted that the findings in one small area of the world may not be relevant to other areas of either the developed or developing world.

We note that the retrospective data analyzed here were collected at a single point in time, and that it will be important to study the development of LOC prospectively through the childhood of the offspring of these men and their partners to verify and understand these results. The plans for such a study are in hand.

## Data availability

In order to preserve confidentiality of the participants it is important that the ALSPAC access rules are taken into account. The ALSPAC study website contains details of all the data that are available through a fully searchable data dictionary: http://www.bristol.ac.uk/alspac/researchers/data-access/data-dictionary/.

Data can be obtained by bona fide researchers after application to the ALSPAC Executive Committee ( http://www.bristol.ac.uk/alspac/researchers/access/).

## Supplementary material

**Supplementary File 1 – Document containing Supplementary Tables 1-11**

.

Table S1. Distribution of the demographic variables concerning the men in the study.

Table S2. Backwards step-wise logistic regression of the man’s locus of control score (≥4 versus <4) in regard to his mother’s demographic features.

Table S3. Backwards step-wise logistic regression of the man’s locus of control score (≥4 versus <4): his father.

Table S4. Backwards step-wise logistic regression of the man’s locus of control score (≥4 versus <4): his early childhood.

Table S5a. Backwards step-wise logistic regression of the man’s locus of control score (≥4 versus <4): his mid-childhood.

Table S5b. Backwards step-wise logistic regression of the man’s locus of control score (≥4 versus <4): his adolescence.

Table S6. Backwards step-wise logistic regression of the man’s locus of control score (≥4 versus <4): his childhood and adolescence.

Table S7. Backwards step-wise logistic regression of the man’s locus of control score (≥4 versus <4): his social environment in childhood.

Table S8. Backwards step-wise logistic regression of the man’s locus of control score (≥4 versus <4): Traumas in his childhood.

Table S9. Backwards step-wise logistic regression of the man’s locus of control score (≥4 versus <4): his childhood traumas and social/medical environment.

Table S10. The childhood and adolescence of father, including childhood traumas and social environment.

Table S11. The childhood and adolescence of father, including childhood traumas, social environment and details of his parents.
