# Supplementary material for: Are the early childhood antecedents of men’s external locus of control similar to those of their female partners?
Source: Wellcome Open Res. 2021 Feb 2;3:28. Originally published 2018 Mar 15. [Version 2] doi: 10.12688/wellcomeopenres.14098.2 (PMC6182670; doi:10.12688/wellcomeopenres.14098.2)
Supplement: Supplementary file 1 [file wellcomeopenres-3-18294-s0000.tgz › ccba4105-a40f-457d-967f-6b3d8be81c00.docx]

Supplementary Tables

**S1 Table. Distribution of the demographic variables concerning the men in the study**

| **VARIABLE** | **PROPORTION IN THE STUDY** | **COMMENTS** |
| --- | --- | --- |
| **Age at questionnaire completion** | |  |
| <20 | 1.2% | Mode 28 |
| 20-24 | 10.3% | Median 30 |
| 25-29 | 33.8% | Mean 30.6; SD 5.7 |
| 30-34 | 33.7% | Range 15 – 65 |
| 35 or more | 21.0% |  |
| **Educational qualifications** |  |  |
| University degree | 19.5% |  |
| **Marital status** |  |  |
| Married | 81.8% |  |
| Separated/widowed/divorced | 4.4% |  |
| Never married | 13.8% |  |
| **Length of time lived in Avon** |  |  |
| < 5 years | 16.8% |  |
| 5 – 9 years | 11.7% |  |
| 10 years or more | 20.3% |  |
| All his life | 50.9% |  |
| **Current employment status** |  |  |
| Unemployed A | 7.4% | Seeking a job |
| Unemployed B | 7.7% | Unemployed as in education, retired, etc. |
| Employed | 84.9% | Employed either full or part-time |
| **Social class** |  | Based on his latest occupation |
| Non-manual | 55.6% |  |
| Manual | 44.4% |  |

# S2 Table. Backwards step-wise logistic regression of the man’s locus of control score (≥4 versus <4) in regard to his mother’s demographic features.

|  | **Univariable** | | | **Model A** | | **Model B** | |
| --- | --- | --- | --- | --- | --- | --- | --- |
| **Variable** | **N** | **P** | **OR [95% CI]** | **P** | **OR [95% CI]** | **P** | **OR [95% CI]** |
| Mother ever smoked | 7829 | <0.0001 | 1.41 [1.29, 1.55] | 0.837 | 1.03 [0.81, 1.31] | 0.292 | 1.10 [0.92, 1.33] |
| Mother’s education ≥ O-Level | 6022 | <0.0001 | 0.51 [0.46, 0.58] | 0.003 | 0.74 [0.61, 0.90] | <0.0001 | 0.57 [0.50, 0.65] |
| Mother’s year of birth (pb420yr)^a^ | 5341 | <0.0001 | 1.48 [1.38, 1.59] | <0.0001 | 1.44 [1.29, 1.60] | <0.0001 | 1.49 [1.37, 1.62] |
| Mother aged ≥25 at birth of father | 6670 | <0.0001 | 0.70 [0.63, 0.77] | 0.180 | 1.17 [0.93, 1.47] | 0.136 | 1.14 [0.96,1.35] |
| Mother’s social group^b^ | 4155 | <0.0001 | 1.58 [1.46, 1.71] | <0.0001 | 1.41 [1.25, 1.59] | - |  |
| Mother smoked when pregnant with him | 7797 | <0.0001 | 1.56 [1.42, 1.71] | <0.001 | 1.39 [1.17, 1.66] | <0.0001 | 1.50 [1.32, 1.71] |

Total N=2258, GOF = 4.23

Model A: N=2258, GOF = 4.23; Model B: N = 4067, GOF = 3.83
^a^ pre 1925; 1925-1939; 1940-1944; post 1944. ^b^ greater than semi-routine; semi-routine; routine occupations

# S3 Table. Backwards step-wise logistic regression of the man’s locus of control score (≥4 versus <4): his father

|  | **Univariable** | | | **Intra domain** | | |
| --- | --- | --- | --- | --- | --- | --- |
| **Variable** | **N** | **P** | **OR [95% CI]** | **N** | **P** | **OR [95% CI]** |
| Father’s year of birth^a^ | 5127 | <0.0001 | 1.42 [1.33, 1.52] | 3578 | <0.0001 | 1.39 [1.28, 1.51] |
| Father’s education ≥ O-Level | 6045 | <0.0001 | 0.52 [0.47, 0.58] | 3578 | 0.005 | 0.80 [0.68, 0.93] |
| Father ever smoked | 7274 | <0.0001 | 1.34 [1.20, 1.50] | 3578 | 0.002 | 1.30 [1.10, 1.54] |
| Father aged ≥25 at birth of father | 6366 | <0.0001 | 0.71 [0.63, 0.81] | 3578 | 0.051 | 1.25 [1.00, 1.56] |
| Father’s social group^b^ | 7173 | <0.0001 | 1.24 [1.21, 1.27] | 3578 | <0.0001 | 1.18 [1.13, 1.23] |

Total N=3578, GOF = 4.38
^a^ pre 1935; 1935-1939; 1940-1944; post 1944. ^b^ the seven categories

# S4 Table. Backwards step-wise logistic regression of the man’s locus of control score (≥4 versus <4): his early childhood

|  | **Univariable** | | | **Model A** | | **Model B** | |
| --- | --- | --- | --- | --- | --- | --- | --- |
| **Variable** | **N** | **P** | **OR [95% CI]** | **P** | **OR [95% CI]** | **P** | **OR [95% CI]** |
| Born outside Avon | 7856 | <0.0001 | 0.38 [0.35, 0.42] | <0.0001 | 0.43 [0.36, 0.50] | <0.0001 | 0.40 [0.35, 0.45] |
| Was breast fed | 5027 | <0.0001 | 0.62 [0.55, 0.70] | <0.001 | 0.73 [0.61, 0.86] | <0.0001 | 0.68 [0.59, 0.78] |
| Number of older siblings | 4850 | <0.0001 | 1.20 [1.14, 1.27] | 0.034 | 1.09 [1.01, 1.18] | - |  |
| Step-father present in home | 8768 | <0.001 | 2.55 [1.57, 4.13] | 0.352 | 1.62 [0.59, 4.46] | 0.730 | 1.15 [0.51, 2.59] |
| Step-sibling present in home | 8768 | 0.020 | 1.66 [1.08, 2.55] | 0.728 | 0.85 [0.35, 2.09] | 0.995 | 1.00 [0.50, 2.02] |
| Parents divorced/separated | 8768 | <0.0001 | 1.72 [1.35, 2.19] | 0.247 | 1.38 [0.80, 2.38] | 0.002 | 1.93 [1.27, 2.92] |
| Unhappy | 7306 | <0.0001 | 1.36 [1.24, 1.49] | 0.587 | 1.05 [0.87, 1.27] | 0.006 | 1.21 [1.5, 1.38] |

Model A: N=2767, GOF =3.83; Model B: N=4056, GOF = 4.72

# S5a Table. Backwards step-wise logistic regression of the man’s locus of control score (≥4 versus <4): his mid-childhood

#

|  | **Univariable** | | | **Intra domain** | | |
| --- | --- | --- | --- | --- | --- | --- |
| **Variable** | **N** | **P** | **OR [95% CI]** | **N** | **P** | **OR [95% CI]** |
| Father present | 8768 | <0.0001 | 0.68 [0.61, 0.77] | 8188 | <0.0001 | 0.55 [0.47, 0.63] |
| Stepfather present | 8768 | <0.0001 | 1.74 [1.34, 2.26] | 8188 | 0.241 | 0.84 [0.62, 1.13] |
| Mother’s partner present | 8768 | <0.0001 | 2.86 [1.72, 4.75] | 8188 | 0.016 | 1.98 [1.14, 3.45] |
| Step-sibling present | 8768 | <0.0001 | 2.15 [1.51, 3.06] | 8188 | 0.174 | 1.29 [0.89, 1.87] |
| Parents divorced/separated | 8768 | <0.0001 | 1.92 [1.57, 2.35] | 8188 | 0.056 | 1.25 [0.99, 1.57] |
| Father died | 8768 | 0.039 | 1.51 [1.02, 2.25] | 8188 | 0.325 | 1.23 [0.81, 1.86] |
| Unhappy | 8358 | <0.0001 | 1.33 [1.25, 1.42] | 8188 | <0.0001 | 1.26 [1.18, 1.35] |
| Smoked regularly | 8525 | <0.0001 | 3.34 [2.24, 4.97] | 8188 | <0.0001 | 3.33 [2.17, 5.12] |

Total N=8188, GOF = 1.78

# S5b Table. Backwards step-wise logistic regression of the man’s locus of control score (≥4 versus <4): his adolescence

|  | **Unadjusted** | | | **Mutually adjusted** | | |
| --- | --- | --- | --- | --- | --- | --- |
| **Variable** | **N** | **P** | **OR [95% CI]** | **N** | **P** | **OR [95% CI]** |
| Father in household | 8768 | <0.0001 | 0.69 [0.62, 0.77] | 8279 | <0.0001 | 0.56 [0.49, 0.63] |
| Brother in household | 8768 | 0.014 | 1.11 [1.02, 1.21] | 8279 | 0.044 | 1.10 [1.00, 1.20] |
| Sister in household | 8768 | 0.001 | 1.15 [1.06, 1.25] | 8279 | 0.005 | 1.14 [1.04, 1.24] |
| Stepfather in household | 8768 | <0.0001 | 1.56 [1.26, 1.93] | 8279 | 0.050 | 0.78 [0.61, 1.00] |
| Step-brother in household | 8768 | 0.026 | 1.45 [1.04, 2.01] | 8279 | 0.961 | 0.99 [0.70, 1.40] |
| Step-sister in household | 8768 | 0.009 | 1.61 [1.13, 2.29] | 8279 | 0.673 | 1.08 [0.74, 1.58] |
| Mother’s partner in household | 8768 | 0.002 | 1.80 [1.25, 2.59] | 8279 | 0.170 | 1.33 [0.89, 1.99] |
| Parents divorced/separated | 8768 | <0.0001 | 1.64 [1.30, 2.07] | 8279 | 0.802 | 1.03 [0.80, 1.34] |
| Started smoking regularly | 8525 | <0.0001 | 2.27 [1.95, 2.64] | 8279 | <0.0001 | 2.23 [1.90, 2.61] |
| Unhappy | 8453 | <0.0001 | 1.19 [1.13, 1.25] | 8279 | <0.0001 | 1.14 [1.08, 1.20] |

Total N=8279, GOF = 2.21

**S6 Table. Backwards step-wise logistic regression of the man’s locus of control score (≥4 versus <4): his childhood and adolescence**

|  | **Unadjusted** | | | **Mutually adjusted** | | |
| --- | --- | --- | --- | --- | --- | --- |
| **Variable** | **N** | **P** | **OR [95% CI]** | **N** | **P** | **OR [95% CI]** |
| ***Early childhood*** |  |  |  |  |  |  |
| Born outside Avon | 7856 | <0.0001 | 0.38 [0.35, 0.42] | 4498 | <0.0001 | 0.39 [0.35, 0.45] |
| Was breast fed | 5027 | <0.0001 | 0.62 [0.55, 0.70] | 4498 | <0.0001 | 0.69 [0.60, 0.79] |
| Parents divorced/separated | 8768 | <0.0001 | 1.72 [1.35, 2.19] | 4498 | 0.717 | 0.92 [0.60, 1.43] |
| Unhappy | 7306 | <0.0001 | 1.36 [1.24, 1.49] | 4032 | 0.160 | 0.88 [0.74, 1.05] |
| ***Mid-childhood*** |  |  |  |  |  |  |
| Father present | 8768 | <0.0001 | 0.68 [0.61,0.77] | 4498 | 0.006 | 0.68 [0.52,0.90] |
| Mother’s partner present | 8768 | <0.0001 | 2.86 [1.72, 4.75] | 4498 | 0.033 | 2.47 [1.08, 5.66] |
| Unhappy | 8358 | <0.0001 | 1.33 [1.25, 1.42] | 4498 | <0.0001 | 1.26 [1.15, 1.39] |
| Started smoking regularly | 8525 | <0.0001 | 3.34 [2.24, 4.97] | 4498 | <0.0001 | 3.76 [1.93, 7.30] |
| ***Adolescence*** |  |  |  |  |  |  |
| Father in household | 8768 | <0.0001 | 0.69 [0.62, 0.77] | 4498 | 0.031 | 0.76 [0.59, 0.98] |
| Brother in household | 8768 | 0.014 | 1.11 [1.02, 1.21] | 4498 | 0.020 | 1.16 [1.02, 1.32] |
| Sister in household | 8768 | 0.001 | 1.15 [1.06, 1.25] | 4498 | 0.075 | 1.12 [0.99, 1.28] |
| Started smoking regularly | 8525 | <0.0001 | 2.27 [1.95, 2.64] | 4498 | <0.0001 | 2.13 [1.71, 2.66] |
| Unhappy | 8453 | <0.0001 | 1.19 [1.13, 1.25] | 4493 | 0.128 | 1.08 [0.98, 1.18] |

Total N=4498, GOF = 7.16

# S7 Table. Backwards step-wise logistic regression of the man’s locus of control score (≥4 versus <4): his social environment in childhood

|  | **Unadjusted** | | | **Mutually adjusted** | | |
| --- | --- | --- | --- | --- | --- | --- |
| **Social environment** | **N** | **P** | **OR [95% CI]** | **N** | **P** | **OR [95% CI]** |
| Attended special school | 8768 | <0.0001 | 2.76 [2.10, 3.63] | 7957 | <0.0001 | 2.38 [1.77, 3.21] |
| Attended child psychiatrist | 8768 | <0.001 | 1.53 [1.21, 1.93] | 7957 | 0.399 | 1.12 [0.86, 1.46] |
| Had speech therapy | 8768 | 0.007 | 1.38 [1.09, 1.74] | 7957 | 0.074 | 1.26 [0.98, 1.61] |
| Was in care | 8299 | <0.0001 | 1.83 [1.40, 2.37] | 7712 | 0.308 | 0.83 [0.59, 1.18] |
| Lived with grandparents | 8490 | <0.0001 | 1.57 [1.31, 1.89] | 7957 | 0.062 | 1.21 [0.99, 1.49] |
| Lived with other relatives | 8490 | <0.0001 | 1.70 [1.37, 2.12] | 7957 | 0.089 | 1.23 [0.97, 1.57] |
| Lived with friends | 8490 | <0.0001 | 1.64 [1.30, 2.07] | 7957 | 0.136 | 1.22 [0.94, 1.58] |
| Lives with foster parents | 8490 | <0.001 | 2.40 [1.46, 3.94] | 7957 | 0.030 | 1.89 [1.06, 3.35] |
| Went to boarding school | 8379 | <0.0001 | 0.57 [0.47, 0.68] | 7957 | <0.0001 | 0.49 [0.40, 0.60] |
| Stayed in children’s home | 8352 | <0.0001 | 2.28 [1.59, 3.27] | 7950 | 0.884 | 0.97 [0.61, 1.54] |
| Stayed in custody | 8325 | <0.0001 | 5.75 [3.97, 8.33] | 7957 | <0.0001 | 5.01 [3.31, 7.58] |
| Left home before age 18 | 8443 | <0.0001 | 1.39 [1.25, 1.55] | 7957 | <0.001 | 1.23 [1.09, 1.39] |
| Mother rarely/never stable | 8298 | <0.0001 | 2.13 [1.69, 2.67] | 7826 | 0.082 | 0.75 [0.55, 1.04] |
| Father rarely/never stable | 7940 | <0.0001 | 2.33 [1.95, 2.78] | 7498 | 0.148 | 1.28 [0.92, 1.80] |
| Home was unstable | 8438 | <0.0001 | 1.84 [1.64, 2.06] | 7957 | <0.0001 | 1.52 [1.34, 1.72] |
| Maternal care score | 8768 | <0.0001 | 0.73 [0.70, 0.77] | 7957 | <0.0001 | 0.80 [0.76, 0.85] |

Total N=7957, GOF = 3.19

# S8 Table. Backwards step-wise logistic regression of the man’s locus of control score (≥4 versus <4): Traumas in his childhood

|  | **Unadjusted** | | | **Mutually adjusted** | | |
| --- | --- | --- | --- | --- | --- | --- |
| **Traumatic event** | **N** | **P** | **OR [95% CI]** | **N** | **P** | **OR [95% CI]** |
| Parent died | 8062 | 0.010 | 1.28 [1.06, 1.54] | 8062 | 0.005 | 1.32 [1.09, 1.59] |
| Sibling died | 8062 | 0.005 | 1.42 [1.11, 1.81] | 8062 | 0.024 | 1.34 [1.04, 1.72] |
| Relative died | 8062 | <0.0001 | 0.79 [0.72, 0.86] | 8062 | <0.001 | 0.83 [0.75, 0.91] |
| Friend died | 8062 | 0.002 | 1.18 [1.06, 1.31] | 8062 | <0.001 | 1.24 [1.11, 1.39] |
| Parent hospitalised | 8062 | <0.0001 | 0.79 [0.73, 0.87] | 8062 | <0.0001 | 0.83 [0.75, 0.91] |
| Admitted to hospital | 8062 | <0.0001 | 0.83 [0.76, 0.90] | 8062 | <0.0001 | 0.82 [0.75, 0.90] |
| Sibling hospitalised | 8062 | 0.005 | 0.86 [0.78, 0.96] | 8062 | 0.174 | 0.93 [0.83, 1.03] |
| Had a serious accident | 8062 | <0.001 | 1.33 [1.14, 1.55] | 8062 | <0.0001 | 1.39 [1.18, 1.63] |
| Parent imprisoned | 8062 | <0.0001 | 3.79 [2.37, 6.07] | 8062 | <0.0001 | 2.78 [1.72, 4.51] |
| Physically abused by parent | 8062 | <0.0001 | 1.74 [1.41, 2.14] | 8062 | 0.001 | 1.44 [1.16, 1.80] |
| Parents separated | 8062 | <0.0001 | 1.79 [1.58, 2.03] | 8062 | <0.0001 | 1.69 [1.48, 1.92] |
| Parents divorced | 8062 | <0.0001 | 1.81 [1.58, 2.07] | 8062 | 0.090 | 1.25 [0.97, 1.60] |
| Parent remarried | 8062 | <0.0001 | 1.75 [1.51, 2.04] | 8062 | 0.085 | 1.19 [0.98, 1.45] |
| Parent emotionally cruel to him | 8062 | 0.019 | 1.23 [1.03, 1.46] | 8062 | 0.100 | 0.84 [0.68, 1.03] |
| Parents had serious arguments | 8062 | <0.001 | 1.19 [1.08, 1.31] | 8062 | 0.492 | 1.04 [0.93, 1.16] |
| Discovered he was adopted | 8062 | 0.009 | 1.50 [1.11, 2.04] | 8062 | 0.012 | 1.49 [1.09, 2.04] |
| Family moved to new district | 8062 | <0.0001 | 0.77 [0.70, 0.84] | 8062 | <0.0001 | 0.74 [0.67, 0.82] |
| Family’s finances worsened | 8062 | 0.001 | 1.22 [1.08, 1.38] | 8062 | 0.175 | 1.09 [0.96, 1.25] |

Total N=8062, GOF = 2.34

# S9 Table. Backwards step-wise logistic regression of the man’s locus of control score (≥4 versus <4): his childhood traumas and social/medical environment

|  | **Unadjusted** | | | **Mutually adjusted** | | |
| --- | --- | --- | --- | --- | --- | --- |
| **Variable** | **N** | **P** | **OR [95% CI]** | **N** | **P** | **OR [95% CI]** |
| *Life Events* |  |  |  |  |  |  |
| Parent died | 8062 | 0.010 | 1.28 [1.06, 1.54] | 7382 | 0.029 | 1.26 [1.02, 1.54] |
| Sibling died | 8062 | 0.005 | 1.42 [1.11, 1.81] | 7382 | 0.041 | 1.32 [1.01, 1.73] |
| Relative died | 8062 | <0.0001 | 0.79 [0.72, 0.86] | 7382 | <0.001 | 0.82 [0.74, 0.91] |
| Friend died | 8062 | 0.002 | 1.18 [1.06, 1.31] | 7382 | 0.001 | 1.22 [1.08, 1.37] |
| Parent hospitalised | 8062 | <0.0001 | 0.79 [0.73, 0.87] | 7382 | <0.0001 | 0.81 [0.73, 0.89] |
| Admitted to hospital | 8062 | <0.0001 | 0.83 [0.76, 0.90] | 7382 | <0.001 | 0.82 [0.75, 0.91] |
| Had a serious accident | 8062 | <0.001 | 1.33 [1.14, 1.55] | 7382 | <0.001 | 1.36 [1.15, 1.62] |
| Parent imprisoned | 8062 | <0.0001 | 3.79 [2.37, 6.07] | 7382 | 0.005 | 2.09 [1.26, 3.49] |
| Physically abused by parent | 8062 | <0.0001 | 1.74 [1.41, 2.14] | 7382 | 0.746 | 0.96 [0.75, 1.23] |
| Parents separated | 8062 | <0.0001 | 1.79 [1.58, 2.03] | 7382 | <0.0001 | 1.40 [1.21, 1.62] |
| Discovered he was adopted | 8062 | 0.009 | 1.50 [1.11, 2.04] | 7382 | 0.093 | 1.33 [0.95, 1.85] |
| Family moved to new district | 8062 | <0.0001 | 0.77 [0.70, 0.84] | 7382 | <0.0001 | 0.72 [0.65, 0.80] |
|  |  |  |  |  |  |  |
| *Social Environment* |  |  |  |  |  |  |
| Attended special school | 8768 | <0.0001 | 2.76 [2.10, 3.63] | 7382 | <0.0001 | 2.29 [1.68, 3.13] |
| Lives with foster parents | 8490 | <0.001 | 2.40 [1.46, 3.94] | 7382 | 0.034 | 1.94 [1.05, 3.57] |
| Went to boarding school | 8379 | <0.0001 | 0.57 [0.47, 0.68] | 7382 | <0.0001 | 0.54 [0.44, 0.67] |
| Stayed in custody | 8325 | <0.0001 | 5.75 [3.97, 8.33] | 7382 | <0.0001 | 4.12 [2.68, 6.34] |
| Left home before age 18 | 8443 | <0.0001 | 1.39 [1.25, 1.55] | 7382 | 0.013 | 1.18 [1.04, 1.34] |
| Home was unstable | 8438 | <0.0001 | 1.84 [1.64, 2.06] | 7382 | <0.0001 | 1.34 [1.16, 1.53] |
| Maternal care score | 8768 | <0.0001 | 0.73 [0.70, 0.77] | 7382 | <0.0001 | 0.81 [0.76, 0.86] |

Total N=7382, GOF = 4.56

# S10 Table. The childhood and adolescence of father, including childhood traumas and social environment

|  | **Unadjusted** | | | **Mutually adjusted** | | |
| --- | --- | --- | --- | --- | --- | --- |
| **Features of childhood** | **N** | **P** | **OR [95% CI]** | **N** | **P** | **OR [95% CI]** |
| *In Infancy* |  |  |  |  |  |  |
| Born outside Avon | 7856 | <0.0001 | 0.38 [0.35, 0.42] | 4369 | <0.0001 | 0.40 [0.35, 0.45] |
| Was breast fed | 5027 | <0.0001 | 0.62 [0.55, 0.70] | 4369 | <0.0001 | 0.72 [0.62, 0.82] |
| *Mid-Childhood* |  |  |  |  |  |  |
| Father present | 8768 | <0.0001 | 0.68 [0.61, 0.77] | 4369 | <0.0001 | 0.64 [0.51, 0.79] |
| Mother’s partner present | 8768 | <0.0001 | 2.86 [1.72, 4.75] | 4369 | 0.019 | 2.89 [1.19, 7.02] |
| Unhappy | 8358 | <0.0001 | 1.33 [1.25, 1.42] | 4326 | 0.530 | 1.04 [0.93, 1.16] |
| Smoked regularly by age 11 | 8525 | <0.0001 | 3.34 [2.24, 4.97] | 4369 | <0.001 | 3.77 [1.87, 7.60] |
| *Adolescence* |  |  |  |  |  |  |
| Father in household | 8768 | <0.0001 | 0.69 [0.62, 0.77] | 4369 | 0.129 | 0.81 [0.62, 1.06] |
| Brother in household | 8768 | 0.014 | 1.11 [1.02, 1.21] | 4369 | 0.051 | 1.14 [1.00, 1.30] |
| Started smoking regularly after 11 | 8525 | <0.0001 | 2.27 [1.95, 2.64] | 4369 | <0.0001 | 2.09 [1.66, 2.62] |
| *Life Events* |  |  |  |  |  |  |
| Parent died | 8062 | 0.010 | 1.28 [1.06, 1.54] | 4369 | 0.081 | 1.31 [0.97, 1.78] |
| Sibling died | 8062 | 0.005 | 1.42 [1.11, 1.81] | 4369 | 0.231 | 1.24 [0.87, 1.78] |
| Relative died | 8062 | <0.0001 | 0.79 [0.72, 0.86] | 4369 | 0.545 | 0.96 [0.83, 1.10] |
| Friend died | 8062 | 0.002 | 1.18 [1.06, 1.31] | 4369 | 0.046 | 1.17 [1.00, 1.37] |
| Parent hospitalised | 8062 | <0.0001 | 0.79 [0.73, 0.87] | 4369 | 0.003 | 0.82 [0.72, 0.93] |
| Admitted to hospital | 8062 | <0.0001 | 0.83 [0.76, 0.90] | 4369 | 0.013 | 0.84 [0.74, 0.96] |
| Had a serious accident | 8062 | <0.001 | 1.33 [1.14, 1.55] | 4369 | 0.023 | 1.31 [1.04, 1.65] |
| Parent imprisoned | 8062 | <0.0001 | 3.79 [2.37, 6.07] | 4369 | 0.601 | 1.23 [0.56, 2.68] |
| Parents separated | 8062 | <0.0001 | 1.79 [1.58, 2.03] | 4369 | 0.076 | 1.21 [0.98, 1.50] |
| Family moved to new district | 8062 | <0.0001 | 0.77 [0.70, 0.84] | 4369 | 0.131 | 0.90 [0.78, 1.03] |
| *Social Environment* |  |  |  |  |  |  |
| Attended special school | 8768 | <0.0001 | 2.76 [2.10, 3.63] | 4369 | 0.003 | 1.83 [1.23, 2.73] |
| Lived with foster parents | 8490 | <0.001 | 2.40 [1.46, 3.94] | 4337 | 0.250 | 1.83 [0.65, 5.12] |
| Went to boarding school | 8379 | <0.0001 | 0.57 [0.47, 0.68] | 4369 | 0.022 | 0.72 [0.54, 0.95] |
| Stayed in custody | 8325 | <0.0001 | 5.75 [3.97, 8.33] | 4369 | <0.001 | 3.02 [1.71, 5.32] |
| Left home before age 18 | 8443 | <0.0001 | 1.39 [1.25, 1.55] | 4313 | 0.132 | 1.15 [0.96, 1.37] |
| Home was unstable | 8438 | <0.0001 | 1.84 [1.64, 2.06] | 4369 | <0.0001 | 1.60 [1.33, 1.93] |
| Maternal care score | 8768 | <0.0001 | 0.73 [0.70, 0.77] | 4369 | <0.0001 | 0.78 [0.72, 0.85] |

Total N=4369, GOF=9.22

# Supplementary Table 11. The childhood and adolescence of father, including childhood traumas, social environment and details of his parents

|  | **Unadjusted** | | | **Mutually adjusted** | | |
| --- | --- | --- | --- | --- | --- | --- |
| **Features of parents and childhood** | **N** | **P** | **OR [95% CI]** | **N** | **P** | **OR [95% CI]** |
| *Parental Characteristics* |  |  |  |  |  |  |
| Mother’s education ≥O-Level | 6022 | <0.0001 | 0.51 [0.46, 0.58] | 2346 | 0.100 | 0.84 [0.69, 1.03] |
| Mother’s year of birth | 5341 | <0.0001 | 1.48 [1.38, 1.59] | 2939 | <0.0001 | 1.32 [1.19, 1.47] |
| Mother smoked in pregnancy | 7797 | <0.0001 | 1.56 [1.42, 1.71] | 2939 | 0.029 | 1.20 [1.02, 1.42] |
| Father’s social group | 7173 | <0.0001 | 1.24 [1.21, 1.27] | 2939 | <0.0001 | 1.16 [1.11, 1.21] |
| *In Infancy* |  |  |  |  |  |  |
| Born outside Avon | 7856 | <0.0001 | 0.38 [0.35, 0.42] | 2939 | <0.0001 | 0.51 [0.43, 0.61] |
| Was breast fed | 5027 | <0.0001 | 0.62 [0.55, 0.70] | 2939 | 0.003 | 0.77 [0.65, 0.91] |
| *In Mid-Childhood* |  |  |  |  |  |  |
| Father present | 8768 | <0.0001 | 0.68 [0.61, 0.77] | 2939 | 0.117 | 0.78 [0.57, 1.06] |
| Mother’s partner present | 8768 | <0.0001 | 2.86 [1.72, 4.75] | 2939 | 0.264 | 2.32 [0.53, 10.13] |
| Smoked regularly by age 11 | 8525 | <0.0001 | 3.34 [2.24, 4.97] | 2939 | 0.003 | 4.24 [1.63, 11.03] |
| *In adolescence* |  |  |  |  |  |  |
| Started smoking regularly after 11 | 8525 | <0.0001 | 2.27 [1.95, 2.64] | 2939 | <0.001 | 1.77 [1.32, 2.38] |
| *Traumatic Events* |  |  |  |  |  |  |
| Friend died | 8062 | 0.002 | 1.18 [1.06, 1.31] | 2939 | 0.032 | 1.23 [1.02, 1.49] |
| Parent hospitalised | 8062 | <0.0001 | 0.79 [0.73, 0.87] | 2939 | 0.869 | 0.99 [0.84, 1.16] |
| Admitted to hospital | 8062 | <0.0001 | 0.83 [0.76, 0.90] | 2939 | 0.323 | 0.92 [0.79, 1.08] |
| Had a serious accident | 8062 | <0.001 | 1.33 [1.14, 1.55] | 2939 | 0.640 | 1.07 [0.81, 1.42] |
| *Social Environment* |  |  |  |  |  |  |
| Attended special school | 8768 | <0.0001 | 2.76 [2.10, 3.63] | 2939 | 0.009 | 1.93 [1.18, 3.15] |
| Went to boarding school | 8379 | <0.0001 | 0.57 [0.47, 0.68] | 2936 | 0.276 | 0.82 [0.58, 1.17] |
| Stayed in custody | 8325 | <0.0001 | 5.75 [3.97, 8.33] | 2939 | 0.012 | 2.53 [1.22, 5.23] |
| Home was unstable | 8438 | <0.0001 | 1.84 [1.64, 2.06] | 2939 | <0.0001 | 1.76 [1.38, 2.25] |
| Maternal care score | 8768 | <0.0001 | 0.73 [0.70, 0.77] | 2939 | <0.0001 | 0.78 [0.71, 0.87] |

Total N=2939, GOF = 9.87
