# Supplementary material for: Are the early childhood antecedents of men’s external locus of control similar to those of their female partners?
Source: Wellcome Open Res. 2021 Feb 2;3:28. Originally published 2018 Mar 15. [Version 2] doi: 10.12688/wellcomeopenres.14098.2 (PMC6182670; doi:10.12688/wellcomeopenres.14098.2)
Supplement: Peer review history

# Peer review: Reviewer response for version 1

**DOI:** 10.21956/wellcomeopenres.15334.r33862

**Published:** 2018-10-10

**Authors:** Dieter Wolke

**Affiliations:** 1 Department of Psychology and Warwick Medical School, University of Warwick, Coventry, UK

Locus of control refers to peoples generalized expectancies about the origin of rewards (e.g. success) and punishment (e.g. failure, bad things happening to them) in the world. As Golding et al. review locus of control does differ from self-efficacy and attribution of causes, however, there is some overlap. Locus of control is a moderately stable individual difference. However, there is still some uncertainty of why some people have external or internal control expectations. This study investigated a large number of men while their partners were expecting a child. This was possibly as part of the Avon Longitudinal Study of Parents and Children (ALSPAC), one of the leading pregnancy to adulthood cohort studies in the world. The men completed a number of questionnaires about their family of origin and their life course behaviour (e.g. such as smoking). The same scales were also completed by the pregnant women and the results have been previously reported by the same group.

 The statistical approach, a theory free exosome approach in hierarchical steps using backward logistic regression is well described. In the final model, after all significant predictor variables were controlled for each other, 12 variables survived for men compared to 20 variables in the previous analysis of women. The results are clearly presented and Table 10 provides a nice comparative summary between the findings on men and women.

 Some of the findings are consistent with previous literature in smaller samples such that more positive maternal care and father presence or breastfeeding are related to less external control while starting to smoke early, having problems in childhood/adolescence which were gendered (e.g. for men: custody; females attended child psychiatrist) were found to be related to more external locus of control. These are easily interpreted as done by the authors that good parenting helps developing internal locus of control and that stresses may be related to external locus of control. However, there are few results that do not neatly fit into this picture of how locus of control develops. It is puzzling that having a parent or oneself who was hospitalized, being born outside Avon, being sent to boarding school or having had a relative die, being physically abused by a parent or having a mentally ill parent (in women) led to more internal locus of control. All of these are potential moderate to severe stressors and it seems that having to master these stresses, i.e. learn coping mechanisms, also increases the belief that the rewards are under once own control. Similar to what has been found in resiliency research, it may be important that children and adolescents are exposed to moderate stressors to learn of how to deal with them and bounce back. Similarly, as shown here it may foster internal locus of control. This would also fit with Baumrind’s concept of tough love: clear rules and exposure to challenges within warm and supportive parenting.

 Alternatively, it may be that those who experienced less good maternal care may have particularly benefited from staying elsewhere, boarding school or moving into a different environment. This could be detected by allowing for statistical interaction terms (e.g. maternal care x boarding school) or subgroup analysis, perhaps something that could be explored. Furthermore, self-rated locus of control provides information about general expectancy, however, it does not tell us how realistic it is or whether it is related to actual rewards. It would be interesting to know whether a higher internal control expectancy in those who had adverse experiences does also reap the same rewards as in those who had mainly positive parenting as a precursor?

 A limitation of the study as acknowledged by the authors is that it is based on retrospective data. Another issue not discussed by the authors is that the questions about the family and life course were asked during pregnancy of their future offspring. It has been previously shown that pregnancy is a period of heightened attention to thoughts such as: “How a good a mother will I be? for expecting mothers while expecting fathers worry about: “Will we have enough money to provide?” and “how will the baby affect my relationship with my partner?”. Thus, during pregnancy recall of information may be partly determined by current concerns and thus it may not be surprising that expectant mothers recalled more about their own parenting than men.

 The future for investigating precursors of locus of control is bright for these authors. The ALSPAC study has collected prospectively data on the offspring of the parents included in this study and the previous report on women removing any concerns about retrospective recall. It will allow to look at other variables that were not included in this study such as how sibling or peer relationships which are important socialization drivers in childhood and adolescence, may be related to locus of control development. Finally, there is the exciting prospect of investigating cross-generational transmission of expectancies. Thus, this study has laid the foundation to guide some of the future prospective analyses.

Is the work clearly and accurately presented and does it cite the current literature?

Yes

If applicable, is the statistical analysis and its interpretation appropriate?

Yes

Are all the source data underlying the results available to ensure full reproducibility?

Yes

Is the study design appropriate and is the work technically sound?

Yes

Are the conclusions drawn adequately supported by the results?

Partly

Are sufficient details of methods and analysis provided to allow replication by others?

Yes

Reviewer Expertise:

Developmental psychopathology, developmental psychology

I confirm that I have read this submission and believe that I have an appropriate level of expertise to confirm that it is of an acceptable scientific standard.

# Peer review: Reviewer response for version 1

**DOI:** 10.21956/wellcomeopenres.15334.r33414

**Published:** 2018-07-20

**Authors:** Aleksandra Kostic

**Affiliations:** 1 Faculty of Philosophy, Study Group for Psychology, University of Niš, Niš, Serbia

Over the last decades, numerous studies have shown that the locus of control is a very significant variable which “permeates“ and shapes different aspects of an individual’s life (personality traits, achievement, professional success, social adaptation and the quality of social interactions). In most studies, the authors researched the factors which caused the domination of either internal or external control in children. I consider this research valuable because there are not many studies which have investigated the antecedents of the loci of control in *adult men*, despite the importance of understanding the process of development of the internal, i.e., external control in the members of this population. Since in their earlier study, the same group of researchers discovered a series of independent factors which were connected to the locus of control in women, the results of the new study enabled the comparison of a series of factors which were connected with the development of the locus of control in men. Of course, both studies used the same theoretical and methodological concepts which enabled the comparison of potential similarities in identified factors on the basis of the information about the parents and the childhood of the respondent.  

 The study included a large number of very important variables (five different groups) which were considered as the potential antecedents of the locus of control. Those were the pieces of information about the demographic background of those men’s parents, about the respondents’ births and early childhood (6 years), about the characteristics of their mid-childhood (6–11 years) and adolescence (12–15 years), about possible traumatic events during their childhood, as well as about their social environment during childhood. The researchers also collected certain information about the parents: their level of education, occupation, ethnic background, stability and predictability of their behaviour. Additionally, the researchers gathered the data on the respondents’ own experience of whether their childhood was happy (childhood happiness).

 The answers to the research questions were obtained by applying adequate standard statistical techniques. The results of the study are particularly important due to a large number of respondents who were included in the research and due to the applied research procedure.

 The results of the research indicate that the antecedents of the locus of control in men and women are  common. Similar experiences during childhood with the mother who is warm, supportive and responsive, who breastfeeds and who has a stable behaviour represents a good basis for the development of the internal locus of control of respondents of both male and female genders (the factors have been identified in the earlier study by the same researchers).

 I am very pleased with all of the segments of the presented study: an excellent theoretical framework, good problematization, appropriate methodology, logical results as well as their interpretation. The researchers are aware of the limitations of their study.

Is the work clearly and accurately presented and does it cite the current literature?

Yes

If applicable, is the statistical analysis and its interpretation appropriate?

Yes

Are all the source data underlying the results available to ensure full reproducibility?

Yes

Is the study design appropriate and is the work technically sound?

Yes

Are the conclusions drawn adequately supported by the results?

Yes

Are sufficient details of methods and analysis provided to allow replication by others?

Yes

Reviewer Expertise:

Social psychology field (communication, social interaction, self-perception, social perception, time perspective, recognition of emotion from facial expression, self-control)

I confirm that I have read this submission and believe that I have an appropriate level of expertise to confirm that it is of an acceptable scientific standard.
